# Limited agreement exists between rationale and practice in athletes' supplement use for maintenance of health: a retrospective study

**DOI:** 10.1186/1475-2891-6-34

**Published:** 2007-10-30

**Authors:** Andrea Petróczi, Declan P Naughton, Jason Mazanov, Allison Holloway, Jerry Bingham

**Affiliations:** 1 School of Life Sciences, Faculty of Science, Kingston University, Penrhyn Road, Kingston upon Thames, Surrey KT1 2EE, UK; 2 School of Business, UNSW@ADFA, Northcott Drive, Canberra ACT 2600, Australia; 3 UK Sport, 40 Bernard Street, London, WC1N 1ST, UK

## Abstract

**Background:**

The widespread use of nutritional supplements among athletes is poorly understood. The prevalence of supplement intake and users' knowledge have been researched independently leading to useful, but disconnected, information on supplement use.

**Methods:**

The 'UK Sport 2005 Drug Free Survey' data (*n *= 874) were re-analysed using association [*χ*^2^] and 'strength of association' tests [*φ*], to discover observed incongruencies between self-reported supplement use and the underlying motives. Results are given for test pairs between 'motive for use' [doctor's advice, avoiding sickness, overcoming injuries and enhancement of diet] and each supplement used and these were categorized as strong (*φ *> .7), intermediate (7 <* φ *> .3) and weak (*φ *< .3).

**Results:**

The use of selected supplements varied widely as follows: multivitamin (72.7%), vitamin C (70.4%), echinacea (30.8%), iron (29.8%), magnesium (11.0%) and ginseng (8.3%). Associations with motive were found in 8 of the 10 test pairs which were expected from literature precedents, however only weak associations exist. Of these, four were associated with avoidance of sickness [iron (χ^2 ^= 11.94, p < .001; φ = .15, p = .001), multivitamin (*χ*^2 ^= 6.43, *p *< .001; *φ *= .11, *p *= .011), vitamin C (*χ*^2 ^= 54.67, *p *< .001; *φ *= .32, *p *< .001) and echinacea (*χ*^2 ^= 40.34, *p *< .001; *φ *= .28, *p *< .001)]. The remaining 4 associations were: no time to prepare meals with ginseng (*χ*^2 ^= 7.64, *p *= .006; *φ *= .12, *p *= .006) and multivitamin (*χ*^2 ^= 9.103, *p *= .003; *φ *= .13, *p *= .003); overcoming injuries with magnesium (*χ*^2 ^= 6.99, *p *= .008; *φ *= .11, *p *= .008); doctors' advice and iron (*χ*^2 ^= 35.00, *p *< .001; *φ *= .25, *p *= .001).

**Conclusion:**

These results suggest a lack of understanding regarding supplements and health maintenance, except for vitamin C and echinacea. Furthermore, supplement use is apparently independent of physicians/dieticians' advice, except for iron. This may suggest a widespread circumvention of expert advice in the growing area of supplement use and therefore should be addressed to underscore potential health risks.

## Background

Vitamins, minerals, herbal remedies, traditional Asian remedies, amino acids and other substances to be taken orally are categorized as 'supplements'. Alternative terms include dietary, food or nutritional supplements or ergogenic aids and in the UK are subject to the general provisions of the Food Labelling Regulations 1996, the Food Safety Act 1990, and the Trade Descriptions Act 1968. The absence of compelling regulation is reflected by the lack of requirement to demonstrate efficacy (unless genetically-modified or claimed to be new) prior to marketing coupled to the blanket ban on medical claims. Considerable variation in concentrations, terminology and combinations of supplements, even within the same country, make it extremely difficult to conduct detailed studies commensurate with pharmaceutical industry type clinical trials. Thus, actual consumption and side effect profiles are yet to be fully elucidated.

Global supplement use in athletes is estimated to range from 40 to as high as 88 percent [1-9] with over thirty thousand supplements being commercially-available in the USA [10]. More than 3 million people in the US alone use, or have used, ergogenic supplements, notably even among 14-year olds [11]. Supplement use in the UK is reportedly less (42% for the UK versus 58% for the US in 2004) [12]. Supplement purchase patterns among women in the US were investigated in a small sample (*n *= 51) which revealed that less than 20% of users made purchase decisions based on product information. Notably, 31% of respondents cited convenience shopping where availability, price and quantity were primarily listed among the deciding factors [13].

Despite the numerous studies of supplement use in athletes, there is a paucity of data on relationships with the motives for use and the supplements actually used. Adolescents reported use of nutritional supplements for i) perceived short-term health benefits, ii) prevention of illness, iii) improved immunity, iv) rectifying poor diet, and v) increased sports performance [14]. Furthermore, adolescents tend to use nutritional supplements provided by parents/guardians and consumed without being aware of the potential health risks [14,15]. For athletes, lack of knowledge or misinformation has been established despite numerous sources of information being available [1,16-19]. The main sources of information used are: athletic trainers (39.8%), strength and conditioning coaches (23.7%), whilst dieticians were only used by 14.4% of athletes to get information about the supplements [1]. Among the other sources accounted for the remaining 22.1% were magazines, team physicians and websites. Intriguingly, an inverse relationship between knowledge and use was established: greater knowledge led to less use of nutritional supplements [18].

Clearly the reasons for, and implications of, unsupervised and unrestricted supplement use require further attention. Key understudied parameters are the driving forces that underlie decision making in supplement use in populations with reliance on maintaining excellent health, such as professional or elite athletes. This report provides the first analysis of relationships that exist between supplement use and the rationale for their use in the maintenance of health. The study cohort had access to specialist support staff (e.g. team doctors or nutritionists) to receive medical support for their daily training regime and healthy diet.

## Methods

Survey data (*n *= 874) collected by UK Sport among high performance British athletes were re-analysed for association between the supplement used and the motives for using such substances for health maintenance [20]. Of the 874 respondents, 528 (60%) reported supplement use. Eight survey forms were excluded because of missing values, resulting in *n *= 520 being available for statistical analyses.

Questions used for these analyses from the original UK Sport "Drug Free Sport" survey are: 'Which supplement do you use or have you used?' and 'Why do you use supplements?' The questions were preceded by a general control question regarding supplement use: "Do you use or have used herbal or nutritional supplements?" Response options (a list of supplements and a list of reasons) were provided and athletes were instructed to select as many as apply and answers were coded: yes = 1, no = 0 [20]. For a full list of response options relevant to health maintenance, refer to Tables 1, 2, 3; row labels (supplements) and column headings (reasons). The complete overlap between the general control question and the two probing questions provides reassurance of the validity of the data (i.e. the respondent gave their full consideration when completing the questionnaire). Athletes who claimed to use supplements also answered the two questions probing into the specifics of their supplement use behaviour (i.e. what and why).

**Table 1:** Pairwise association (chi-square test statistics and corresponding p-values) between reason to use and type of supplements used (all athletes who use supplements n = 520), testing H_0_: independence

	No time preparing meal	Avoid sickness	Over-come injury	Doctors' advice
Iron	*χ*^2 ^= *1.798*	*χ*^2 ^= ***11.940***	*χ*^2 ^= 0.350	*χ*^2 ^= ***35.004***
	*p = .180*	*p *= ***.001***	p = .554	*p *<***.001***
Ginseng	*χ*^2 ^= *7.636*	*χ*^2 ^= *0.757*	*χ*^2 ^= 3.238	*χ*^2 ^= 4.055
	*p = .006*	*p = .384*	p = .072	p = .044
Multi Vitamin	*χ*^2 ^= *9.103*	*χ*^2 ^= *6.427*	*χ*^2 ^= 0.970	*χ*^2 ^= 0.190
	*p = .003*	*p = .011*	p = .325	p = .892
Vitamin C	*χ*^2 ^= 0.104	*χ*^2 ^= ***54.671***	*χ*^2 ^= 0.012	*χ*^2 ^= 0.005
	p = .747	*p *<***.001***	p = .912	p = .944
Magnesium	*χ*^2 ^= 1.267	*χ*^2 ^= 0.140	*χ*^2 ^= *6.997*	*χ*^2 ^= 1.883
	p = .260	p = .708	*p = .008*	p = .170
Echinacea	*χ*^2 ^= 1.206	*χ*^2 ^= ***40.343***	*χ*^2 ^= 0.806	*χ*^2 ^= 0.003
	p = .272	*p *<***.001***	p = .369	p = .959

*Statistically significant associations (at p ≤ .001) are in bold.

**Table 2:** Strength of associations (*φ *coefficients and corresponding p-values) between reason to use and type of supplements used (all athletes who use supplements n = 520)

	No time preparing meal	Avoid sickness	Over-come injury	Doctors' advice
Iron	*φ *= *.059*	*φ *= ***.152***	φ = .026	*φ *= ***.259***
	*p = .180*	*p < .001*	p = .554	*p < .001*
Ginseng	*φ *= *.121*	*φ *= *.038*	φ = .079	φ = -.088
	*p = .006*	*p = .394*	p = .072	p = .044
Multi Vitamin	*φ *= *.132*	*φ *= *.111*	φ = .043	φ = -.006
	*p = .003*	*p = .011*	p = .325	p = .812
Vitamin C	φ = .014	*φ *= ***.324***	φ = -.005	φ = .003
	p = .747	*p < .001*	p = .912	p = .944
Magnesium	φ = .044	φ = -.016	*φ *= *.116*	φ = .060
	p = .260	p = .708	*p = .008*	p = .170
Echinacea	φ = .048	*φ *= ***.278***	φ = .039	φ = .002
	p = .272	*p < .001*	p = .369	p = .959

* Statistically significant *φ *coefficients (at *p *≤ .001) are in bold and associations are categorized as strong (*φ *> .7), intermediate (7 <*φ *> .3) and weak (*φ *< .3).

**Table 3:** Relative percentage of congruent answers by rationale for supplement use and supplement used (n = 520)

		No time preparing meals				Avoid sickness			
		
		Yes		No		Yes		No	
Multivitamin	Yes	*84*	*84.8%*	*294*	*69.8%*	*253*	*76.4%*	*125*	*66.1%*
	No	*15*	*15.2%*	*127*	*30.2%*	*78*	*23.6%*	*64*	*33.9%*
		***99***	***100.0%***	***421***	***100.0%***	***331***	***100.0%***	***189***	***100.0%***
Vitamin C	Yes	71	71.7%	295	70.1%	*270*	*81.6%*	*96*	*18.5%*
	No	28	28.3%	126	29.9%	*61*	*18.4%*	*93*	*17.9%*
		**99**	**100.0%**	**421**	**100.0%**	***331***	***100.0%***	***189***	***36.3%***
Magnesium	Yes	14	14.1%	43	10.2%	35	10.6%	22	4.2%
	No	85	85.9%	378	89.8%	296	89.4%	167	32.1%
		**99**	**100.0%**	**421**	**100.0%**	**331**	**100.0%**	**189**	**36.3%**
Echinacea	Yes	35	35.4%	125	29.7%	*134*	*40.5%*	*26*	*13.8%*
	No	64	64.6%	296	70.3%	*197*	*59.5%*	*163*	*86.2%*
		**99**	**100.0%**	**421**	**100.0%**	***331***	***100.0%***	***189***	***100.0%***
Iron	Yes	*35*	*35.4%*	*120*	*28.5%*	*116*	*35.0%*	*39*	*20.6%*
	No	*64*	*64.6%*	*301*	*71.5%*	*215*	*65.0%*	*150*	*79.4%*
		***99***	***100.0%***	***421***	***100.0%***	***331***	***100.0%***	***189***	***100.0%***
Ginseng	Yes	*15*	*15.2%*	*28*	*6.7%*	*30*	*9.1%*	*13*	*6.9%*
	No	*84*	*84.8%*	*393*	*93.3%*	*301*	*90.9%*	*176*	*93.1%*
		***99***	***100.0%***	***421***	***100.0%***	***331***	***100.0%***	***189***	***100.0%***

* 100% in each cell indicates a complete congruence.

The reasons for use and supplements used were selected if the *primary *goal for, or effect of, taking the substance was health related. Health maintenance as a general goal was operationally defined as a combination of health preserving and improving strategies (i.e. avoiding sickness, overcoming injury, balancing inadequate diet resulting from no time to prepare meals and medical advice). Substances were categorised by their effect. In a case, where a supplement may serve health maintenance and performance enhancing reasons (e.g. vitamin C), the supplement was included in the analysis.

Congruency between supplement used and motives for using such substances were detected by Chi-square test of association and the strength of relationship was estimated by calculating *φ *coefficients [21]. Chi-square procedures test for dependence between the two variables (reason and supplement) whereas *φ *coefficients extend this information by estimating the strength of the observed relationship. A *φ *coefficient near +1 implies the athletes responded in agreement (i.e. Yes-Yes or No-No) to both statements. A *φ *coefficient near -1 implies that athletes did not respond in agreement (i.e. Yes-No or No-Yes) to both statements. A *φ *coefficients near 0 imply no association exists between the statements. Significant association is indicated by the *p*-value corresponding to each *chi-square *statistics (*χ*^2^) and *φ *coefficient.

**Figure 1:**
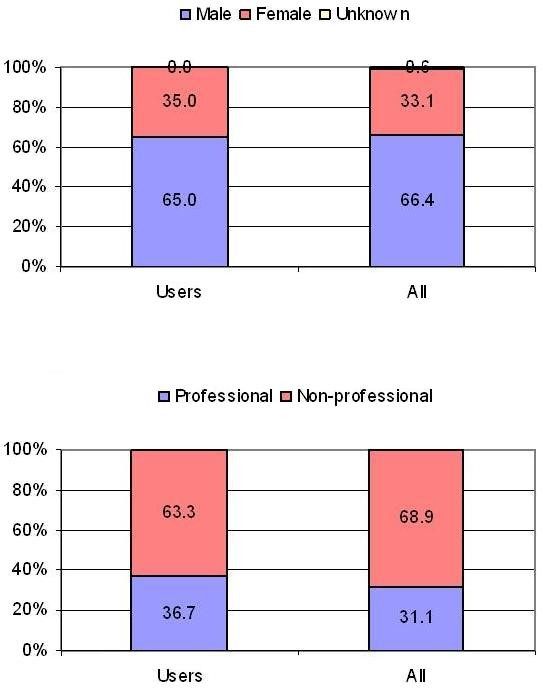
Gender and status distribution in the sample (n = 874) and user sub-sample (n = 520).

**Figure 2:**
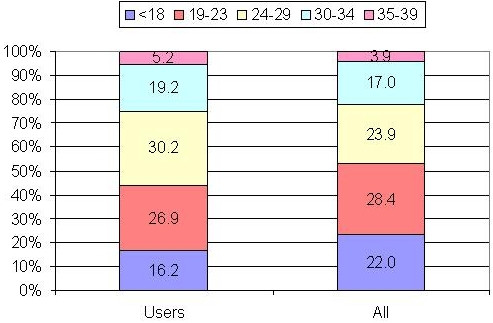
Age distribution in the sample (n = 874) and user sub-sample (n = 520).

Proportions of gender, age and status (professional players vs. lottery funded athletes) roles within the full sample and supplement user sub-sample were compared. Observed frequencies in each sub-sample (i.e. gender, age or professional status) were compared to the corresponding expected frequencies. Expected frequencies for each sub-sample were calculated from the proportion of the sub-sample in the full sample (i.e. the full sample consisted of 66.4% males), under the assumption that the sample composition remained the same, the expected frequency count of males among supplement user is 345 [66.4% of 520]. Chi-square goodness of fit statistics were used to determine whether the discrepancy (if any) was statistically significant. Bar charts were used to illustrate the gender, age group and profile distribution in the sample and in the supplement user cohort (Figures 1 and 2). Statistical analyses were performed using SPSS.14.0.1.

Cells have been identified as 'expected congruency between reasons and actions' based on anecdotal evidence (expected but may be incorrect association) and empirical proofs (expected correct association). The accuracies of the associations were determined using published research on the effects of nutritional supplements [7,14,22-30]. Based on these literature precedents, positive associations (in italics in Tables 1, 2, 3) were expected for the following pairs: multivitamin – *avoiding sickness*, *no time to prepare meals*; vitamin C – *avoiding sickness*; echinacea – *avoiding sickness*; iron – *no time to prepare meals*, *avoiding sickness *and *doctors' advice*; Ginseng – *no time to prepare meals *and *avoiding sickness*.

Note that athletes in the sample were not explicitly asked to give a rationale for using a particular supplement. Congruency was statistically tested between the two answers athletes gave independently by comparing the proportion of concordant pairs to a proportion of concordant pairs expected under the no association condition [31].

## Results

### Sample Characteristics

The 'UK Sport 2005 Drug Free Survey' full sample (*n *= 874) predominantly consisted of male (66.4%) lottery-funded athletes (69%) compared to professional players (31%) with over 30 sports represented. The UK Sport survey uses the term 'Athlete' for lottery-funded non-professionals and 'Players' for professionals in football, cricket, rugby union, rugby league, tennis, ice hockey and basketball. In this paper, we use the term 'athlete' for the combined sample of professional players and lottery-funded athletes together. The majority of respondents (52.3%) were between age 19 and 29 whilst the <18 and 35–39 age groups formed considerable segments (22% and 17%, respectively). Athletes who did not use supplements were eliminated from further analysis, reducing the analysed data set to *n *= 520, which was still higher than the minimum acceptable sample (> 50).

The sample characteristics of the supplement users sub-sample were also investigated and compared to the full sample. Whilst there were observable small differences in the proportion of males and females; professional, non-professional athletes (Figure 1) or by age (Figure 2), the overall difference was not statistically significant for gender (*χ*^2 ^= 0.14, *p *= .71), professional status (*χ*^2 ^= 1.40, *p *= .24) or age (*χ*^2 ^= 7.03, *p *= .533). Thus, it can be concluded that selecting those athletes who reported supplement use did not change the composition of the sample. The strength of association exhibited by these subgroups individually requires further investigation. However, analyses of the roles of gender, age and professional status on supplement use is beyond the scope of this paper.

**Figure 3:**
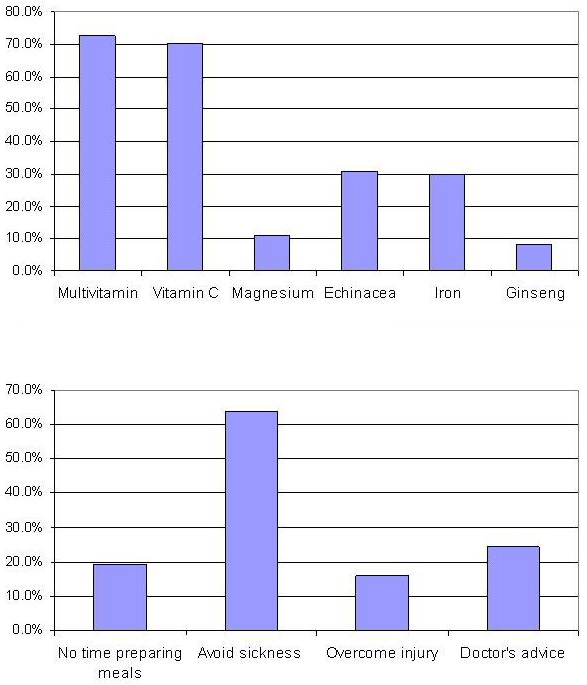
Health related reasons for use and supplement used by high performance athletes (n = 520).

Figure 3 shows that the most popular supplements used were multivitamins and vitamin C (72.7 and 70.4% respectively) with approximately one third of athletes taking echinacea and/or iron (30.8 and 29.8% respectively). A minority used magnesium (11.0%) and ginseng (8.3%). Analyses of the motives behind supplement use for the maintenance of health revealed that the desire to *avoid sickness *predominates (63.6%). A notable cohort of 24.2% indicated the reasons for supplement use as *doctors' advice*, with 19.0% listing *no time to prepare meals *and 16.0% citing *overcoming injury*.

### Associations between reasons and supplements

The prevalence of associations is given in Table 1 with strength of associations in Table 2. Table 1 shows that of the expected associations (10 test pairs, in italics), only 4 were significant at p ≤ .001 level (in bold). No significant associations were observed for iron with *no time to prepare meals *or ginseng with *avoiding sickness*. The strength of these associations, however, varied (Table 2). Of the 10 pairs, only one showed an intermediate association, and was not surprisingly: *avoiding sickness *– vitamin C.

The relatively small *φ *coefficients for the 8 out of 10 associations found in Table 2 warrant further analyses. Tables 3 and 4 show the relative percent of expected congruent behaviour (YY and NN cells). High percentage in these cells indicates good knowledge of nutritional supplements whereas high percentage in the mixed cells (YN and NY) suggests lack of understanding and possible random use of supplements. For example, when an athlete indicates prevention as reason for supplement use, use of vitamin C is expected. Ideally, the YY cell in this case would be 100%. If informed behaviour was operationally defined as observed congruency at least above the upper quartile threshold (75%), only two supplements (vitamin C and multivitamin) reached this threshold.

**Table 4:** Relative percentage of congruent answers by rationale for supplement use and supplement used (n = 520)

		Overcome injury				Doctor's advice			
		
		Yes		No		Yes		No	
Multivitamin	Yes	64	77.1%	314	71.9%	91	72.2%	287	72.8%
	No	19	22.9%	123	28.1%	35	27.8%	107	27.2%
		**83**	**100.0%**	**437**	**100.0%**	**126**	**100.0%**	**394**	**100.0%**
Vitamin C	Yes	58	69.9%	308	70.5%	89	70.6%	277	70.3%
	No	25	30.1%	129	29.5%	37	29.4%	117	29.7%
		**83**	**100.0%**	**437**	**100.0%**	**126**	**100.0%**	**394**	**100.0%**
Magnesium	Yes	*16*	*19.3%*	*41*	*9.4%*	18	14.3%	39	9.9%
	No	*67*	*80.7%*	*396*	*90.6%*	108	85.7%	355	90.1%
		***83***	***100.0%***	***437***	***100.0%***	**126**	**100.0%**	**394**	**100.0%**
Echinacea	Yes	29	34.9%	131	30.0%	39	31.0%	121	30.7%
	No	54	65.1%	306	70.0%	87	69.0%	273	69.3%
		**83**	**100.0%**	**437**	**100.0%**	**126**	**100.0%**	**394**	**100.0%**
Iron	Yes	27	32.5%	128	29.3%	*64*	*50.8%*	*91*	*23.1%*
	No	56	67.5%	309	70.7%	*62*	*49.2%*	*303*	*76.9%*
		**83**	**100.0%**	**437**	**100.0%**	***126***	***100.0%***	***394***	***100.0%***
Ginseng	Yes	11	13.3%	32	7.3%	5	4.0%	38	9.6%
	No	72	86.7%	405	92.7%	121	96.0%	356	90.4%
		**83**	**100.0%**	**437**	**100.0%**	**126**	**100.0%**	**394**	**100.0%**

* 100% in each cell indicates a complete congruence.

## Discussion

In some cases, the motives for use and the supplements used show a great deal of incongruence. This suggested a lack of knowledge or understanding of nutritional supplements' effects, except vitamin C, which was associated, but not strongly with preventing illness (i.e. those who take supplements for health reasons tend to take vitamin C). No other supplement pairing with motive for use revealed either a strong or intermediate association.

Athletes' responses were also inconsistent regarding medical advice informing supplement use. This is a worrying sign considering that about 60% of athletes seem to take supplements (Figure 3), but many apparently do not do so because of medical advice. This is interesting because when asking about individuals providing information and advice regarding doping issues, medical practitioners appeared to be the most common information source [20]. Team doctors were the only group that obtained net positive ratings (net positive ratings were calculated in the UK Sport report by subtracting the number of no-selections of doctors from the number of affirmative answers on the same). Yet, no significant association was found between medical advice and any supplements used except iron.

The benefits and drawbacks of supplement use have received considerable attention in recent years, with conflicting reports frequently appearing in the literature. The complex nature of unregulated supplement use seriously hampers the instigation of studies with robust outcomes commensurate with clinical trial approved procedures. Where reports of the observed safe levels (OSL) of individual supplements exist, they are frequently negated by the practice of intake of levels well beyond the OSL. In addition, the intake of multiple supplements is a common practice which manifests in both a higher number of adverse reactions and in those reactions being more severe [10]. Regarding supplement use in athletes, further parameters under scrutiny include the interaction between medicines and supplements, the source and purity of supplements and the effects of injury or disease [32].

Among all the supplements queried in the survey, vitamin C use can be the most accurately predicted from the reasons given for using such supplements and *vice versa*. Athletes are likely to take a combination of substances and perhaps in large doses. Potentially adverse effects of long-term use of vitamin C (for example as a pro-oxidant at high levels) have been established along with the beneficial effects [33,34]. Reservations therefore apply to even our most commonly used supplements regarding long-term use and appropriate dosages. These reservations concern: 1) an increased health risk to an otherwise healthy population [4,35,36], and 2) the possibility of positive doping tests caused by supplements containing banned substances.

Supplement use is predominant among athletes (62% of the athletes in this sample indicated use). Research shows that athletes are willing to take supplements based on personal recommendation without gathering reliable information about the substance, often obtaining them directly from retailers and internet sites [15,37]. Thus, illegal substances (such as those on the World Anti Doping Agency [WADA] Prohibited List or narcotics) and supplements may reach athletes through the same distribution channels [37,38]. In order to regulate the European market, the European Union issued the Food Supplements Directive 2002/46/EC, which was implemented in the UK in 2003, effective from August 2005 [39]. Under the EU Directive, a so-called 'positive list' has been created listing the allowable vitamins, minerals and permitted chemical forms (sources) of these vitamins and minerals that may be used in food supplements and has been widely criticised for its inconsistent inclusion/exclusion criteria and for the costs involved with adding items to the list [40]. The Food Standards Agency (UK) has successfully rebutted the EU's attempt and, by virtue of the derogation in Article 4.6. of the Directive, which permits the continued use of vitamins and minerals not on the 'positive lists', the UK supplement market will remain semi-regulated at least until 2009 [40]. Unless strong evidence is found for adverse effects, health warnings are therefore not likely to be placed on nutritional supplements [5]. The conundrum is how to obtain the strong evidence in the absence of rigorous regulation which severely limits the validity of data collected.

Accurate and adequate information should be provided to athletes via channels they actually use and in a format they are willing to consider (e.g. ATLAS [41] and ATHENA [42]). Research conducted in various settings uniformly came to the conclusion that coaches are the most influential persons on athletes' behaviour, being perceived by athletes as knowledgeable and credible information sources [1,5,43]. It is imperative that education in supplement use advice should become a required part of the accreditation process for coaches in much the same way as psychologists are sometimes trained in psychopharmacology to allow prescription of relevant psychoactive medication.

Further research would benefit from using a sample drawn from a wider cross-section of the athlete population and should explore in more depth athletes' beliefs and knowledge about the effectiveness of supplements. It would also be valuable to focus on differences between endurance and non-endurance athletes in this respect with an expanded list of supplements. The reasons for taking supplements should be further investigated in relation to psychological factors such as blocked or unattainable goals and the extent to which supplement use is perceived as a transitional milestone on the road to becoming a serious athlete. The conundrum of supplement using behaviour can be overcome through this fundamental deconvolution approach.

## Conclusion

This study provided a sound platform for assessing congruence between athletes' reasons for supplement use and their actual use. Incongruence regarding nutritional supplements and their effects (evidenced by reasons) is alarming. Athletes seem to take supplements without an understanding of the benefits they can offer, or the side effects. With the exception of vitamin C and multivitamins, less than 50% showed congruence between reasons and actions (supplements taken) suggesting that supplements may be used by high performing athletes without a clear, coherent plan.
